# Supplementary material for: Insight on the anatomy, systematic relationships, and age of the Early Cretaceous ankylopollexian dinosaur Dakotadon lakotaensis
Source: PeerJ. 2015 Sep 22;3:e1263. doi: 10.7717/peerj.1263 (PMC4582955; doi:10.7717/peerj.1263)
Supplement: Supplemental Information 1 — All OTUs that were excluded from the final analysis shown in Fig. 10 are noted by ∗. See text for further details. Abbreviations: a, polymorphic coding of 3/4; b, polymorphic coding of 4/5. [file peerj-03-1263-s001.docx]

|  | 1 0 | 2 0 | 3 0 | 4 0 | 5 0 |
| --- | --- | --- | --- | --- | --- |
| *L. diagnosticus* | 00000?0000 | 0000000000 | 00??000000 | 0000??0?00 | 0000000000 |
| *H. foxii* | 00000?0000 | 0000000000 | 00??010000 | 0000?00000 | 0000000000 |
| *M. langdoni* | ?????????? | ?????????? | ?????1???? | ?????????? | 00??12???? |
| *Rhabdodon* sp. | ???????000 | 0000110000 | 00??0?10?? | ?????????? | ?????????? |
| *Z. shqiperorum* | 1??00?0000 | 2110112000 | 00???1???? | ?????????? | ?????????? |
| *Z. robustus* | 10100?0000 | 0100112000 | 00??011001 | 1000??0000 | 0000?0?011 |
| *T. tilletti* | 1?00101000 | 0000110000 | 00??010011 | 1100????10 | 00??000000 |
| *T. dossi* | 1000101??0 | ?0???1000? | 00??010010 | 1100?01010 | 0000000000 |
| *D. altus* | 101?1????0 | 0000110000 | 00??010011 | 1100?01010 | 0000000000 |
| *V. canaliculatus* | ?????????? | ?????????? | ?????????? | ?????????? | ?????????? |
| *D. lettowvorbecki* | 1011101010 | 0000110000 | 00??0?0011 | ??00?????0 | 0000000020 |
| *C. dispar* | 1011101010 | 1000110000 | 00??????11 | 11010????0 | 0000010??? |
| *C. prestwichii* | ?????????? | 10001???00 | ?????1001? | 1?0??????0 | ?000010??? |
| *U. aphanoecetes* | ?????????? | ?0?0110??0 | 00???????? | ?????????? | ?????????? |
| *D. lakotaensis* | 101?101010 | 10?0110??0 | 00??0???11 | 1101101110 | 1100111000 |
| *L. magnidens* | ???????010 | 1000210000 | 00??0100?? | ?????????? | ?????????? |
| *B. dawsoni* | ???????011 | 1000?1000? | 1100?????? | ?????????? | ?????????? |
| *B. yixianensis* | ??1?111?11 | ?0?031?1?0 | 00?1????11 | 101??1111? | 10??122030 |
| *H. scutodens* | ?????????? | 100??11000 | ????0?00?? | ?????011?? | ?00?111030 |
| *T. kerri* | ?????????? | ???????000 | 00??010011 | 1101001110 | 1???1?1030 |
| *O. depressus** | ?????????? | ?????????? | ?????????? | ?????????? | ?????????? |
| *I. fortis* | ???1?????? | ?????????? | ?????????? | ?????????? | ?10?111??? |
| *P. valdearinnoensis* | 0011101010 | 1200411011 | 1110??2111 | 110111??10 | 0112112??? |
| *K. katsuyama* | ?????????? | ?????????? | ?????????? | ?????????0 | 01121121?? |
| *I. bernissartensis* | 1111101011 | 1000311010 | 11000?0011 | 110110111? | 11??122032 |
| *M. atherfieldensis* | 1111101011 | 1000311110 | 11000?0011 | 1101101111 | 1110122032 |
| *A. kurzanovi* | 1110111?11 | ?0?0311011 | 1110010011 | 110??11011 | 11??123130 |
| *E. normani* | ??11111011 | ?0???1111? | 1110022011 | 110110111? | 11??123102 |
| *H. fittoni* | ?????????? | ?????????? | ?????????? | ?????????? | ?????????? |
| *X. yueluni* | 111?111011 | ?0???1011? | 11??022011 | 110110101? | 101?113102 |
| *G. mazongshanensis* | ?????????? | ?????????? | ?????????? | ?????????? | ?????????? |
| *P. gobiensis* | 11?1111011 | 10013111?1 | 11100?201? | 110??1??1? | ?1??12???? |
| *E. caroljonesa* | 11?1111011 | 1001321111 | 1100022011 | 110111??11 | 1110123??? |
| *J. rugoculus* | 1??????011 | 1001311111 | 1100?????? | 1????????? | ?021123??? |

|  | 1 0 | 2 0 | 3 0 | 4 0 | 5 0 |
| --- | --- | --- | --- | --- | --- |
| *P. byrdi* | 11111?1111 | 1001321111 | 1100022011 | 11011???11 | 1010123??? |
| *N. zhugeii* | ?????????? | ?????????? | ?????????? | ?????????? | ?????????? |
| *T. insularis* | ??1?111??0 | ?0???110?? | 21??12?111 | 120131111? | 00??133102 |
| *L. transoxiana* | 1??11?1011 | 0001?11?1? | ????1??0?? | ?????????? | ?0??133??? |
| *G. mongoliensis* | 11?111101? | ?0???11??? | ?????????? | ?????????1 | ?022133102 |
| *B. johnsoni* | 1111121011 | 2011311011 | 11101?2011 | 12012???11 | 1010133102 |
| *S. gilmorei* | ???????111 | 2011311011 | ?11??????? | ?????11??1 | 11??13313? |
| *T. transsylvanicus* | ???????111 | 2011311111 | 21101?2111 | 12012???11 | 1012133??? |
| *C. casuarius* | 2111121?11 | 2011411111 | 2111122111 | 121121111? | 10??133102 |
| *E. annectens* | 2111121111 | 2011411111 | 2111122111 | 1211211111 | 1022133102 |
| *T. asinensis** | ?????????? | ?????????? | ?????????? | ?????????? | ?????????? |
| *C. agilis** | ?????????? | ???1?????? | ?????????? | ?????????? | ????13???? |
| *K. coetzeei** | ?????????? | ?????????? | ?????????? | ?????????? | ?????????? |
| *L. atopus** | ?????????? | ?????????? | ?????????? | ??????11?? | ?0???????? |
| *L. arenatus** | 1????????? | ?????????? | ????01001? | ??0??????? | ?????????? |
| *H. foulkii** | ?????????? | ?????????? | ?????????? | ?????????? | ?????????? |
| *R. suranareae** | ???????011 | 000030?011 | 011??????? | ?????????? | ?????????? |
| *O. hoggii** | ????????10 | 1200100000 | 00???????? | ?????????? | ?????????? |
| *P. weishampeli** | ???????010 | 1001310111 | 1100?????? | ?????????? | ?????????? |
| *P. venenica** | ?????????? | ?????????? | ?????????? | ?????????? | ?????????? |
| *J. meniscus** | ?????????? | ?????????? | ?????????? | ?????????? | ?????????? |
| *C. crichtoni** | ?????????? | ?????????? | ?????????? | ?????????? | ?????????? |
| *F. tetoriensis** | 0011111010 | 1?10302010 | 1100012011 | 11011????0 | ?1??113??? |
| *D. turolensis** | ?????????? | ?????????? | ?????????? | ?????????? | ?????????? |
| *O. nigeriensis** | 21?1101111 | 1000320110 | 0???010011 | 120111101? | ?112122032 |
| *J. yangi* | 1?0?1?1?11 | ?0???1011? | 11??0?001? | 1????1111? | 10??1231?2 |
| *C. leedsi** | ?????????? | ?????????? | ?????????? | ?????????? | ?????????? |
| *C. valdensis** | ?????????? | ?????????? | ?????????? | ?????????? | ?????????? |
| *D. loureiroi** | ?????????? | ?????????? | ?????????? | ?????????? | ?????????? |
| *E. nigeriensis** | ?????????? | ?????????? | ?????????? | ?????????? | ?????????? |
| *G. ericksoni** | ?????????? | ?????????? | ????????11 | ??012???1? | ?????????? |
| NHMUKR 8676* | ?????????? | ?????????? | ?????????? | ?????????? | ?????????? |
| NHMUKR 3741* | ?????????? | ?????????? | ?????????? | ?????????? | ?????????? |
| NHMUKR 1831* | ???????011 | 1000200000 | ?????????? | ?????????? | ?????????? |

|  | 6 0 | 7 0 | 8 0 | 9 0 | 1 0 0 |
| --- | --- | --- | --- | --- | --- |
| *L. diagnosticus* | 0000000000 | ?00?000?00 | 000?0?0000 | 000000??00 | 0??00????0 |
| *H. foxii* | 0000001010 | ?010000000 | 000?000000 | 0000010000 | 1000000000 |
| *M. langdoni* | ????1?1?00 | ?0??0???00 | 000?0????0 | ????????10 | 1?1??????? |
| *Rhabdodon* sp. | ??1??????? | ?????????? | ?????????? | 10000101?? | ?????????? |
| *Z. shqiperorum* | ??101?11?0 | ?01?111?10 | 0???1000?0 | 10000101?? | ????0????0 |
| *Z. robustus* | 0?1??01100 | ?011111?10 | 01??010010 | 1000010110 | 1?110????0 |
| *T. tilletti* | 0?0?3?1010 | ?01?010001 | 0010001010 | 1000010010 | 1000000000 |
| *T. dossi* | 0000??1010 | ?01??10001 | 0?100??0?0 | 1??0????10 | ???000000? |
| *D. altus* | 0?0?0??101 | 0110010000 | 0010001010 | 1000011010 | 1000000000 |
| *V. canaliculatus* | ?????????? | ?????????? | ?????????? | ?????????? | ????0????? |
| *D. lettowvorbecki* | ?0?00?11?1 | 011??10?00 | 00100?1010 | 1000011010 | 1000000000 |
| *C. dispar* | ???0?0???1 | 0110000?00 | 0010011210 | 1000011210 | 1000010000 |
| *C. prestwichii* | ???0?0???? | ????????0? | ????0111?0 | 1000011210 | 100?0????1 |
| *U. aphanoecetes* | ?????????? | ?????????0 | 00100111?0 | 1??0011??? | ???0010001 |
| *D. lakotaensis* | 1??001???? | ??????000? | 00??01?010 | 1000011210 | 100??????? |
| *L. magnidens* | ?????????? | ?????????? | ?????????? | 11000110?? | 1?0?1?100? |
| *B. dawsoni* | ?????????? | ?????????? | ?????????? | 110?0112?? | ?????11??1 |
| *B. yixianensis* | 11??0????? | ??1?1????? | ?????????? | 1110011210 | 100010???1 |
| *H. scutodens* | 111?0?210? | ?00?00000? | ??10?????? | 1?00011?10 | ????1?1001 |
| *T. kerri* | 11????210? | ?00?0????? | ?????????? | 10??01121? | 100??????? |
| *O. depressus** | ?????????? | ?????????? | ?????????? | ?????????? | ?????????? |
| *I. fortis* | ?????????1 | 012001???? | ?????????? | ????01?2?? | 1?01?????1 |
| *P. valdearinnoensis* | ??1??????1 | 0?20000101 | 0010000?11 | 1000011210 | 100??????? |
| *K. katsuyama* | ?????????? | ?????????? | ?????????? | ????????10 | 102??????? |
| *I. bernissartensis* | 112?0?1101 | 012?000001 | 01100???11 | 1100011210 | 1001111011 |
| *M. atherfieldensis* | 1110211101 | 0102000001 | 011?00???1 | 1100011210 | 1001111000 |
| *A. kurzanovi* | 01102?3101 | 010?00??0? | ?????????? | 1100011210 | 102???1001 |
| *E. normani* | 111?2?3101 | 010?00??0? | ??1001???1 | 1100011210 | 102?1?111? |
| *H. fittoni* | ?????????? | ?????????? | ?????????? | ?????????? | ????1????0 |
| *X. yueluni* | 011?2?3101 | 0?1?000101 | ??10?1???0 | 1?00011210 | 102?11???? |
| *G. mazongshanensis* | ?????????1 | ?????????? | ?11??????0 | ?1??01?3?? | 1?3?1????0 |
| *P. gobiensis* | ???0?13101 | 011?00010? | ??100????? | 121111131? | 103?1?1000 |
| *E. caroljonesa* | ???03131?1 | 0112000101 | 0111001210 | 1211111b10 | 103?1?1010 |
| *J. rugoculus* | ?????????1 | 11020????? | ?????????? | 121?11131? | 103?1????? |

|  | 6 0 | 7 0 | 8 0 | 9 0 | 1 0 0 |
| --- | --- | --- | --- | --- | --- |
| *P. byrdi* | ?1?0313101 | 102?0???0? | ?????????? | ?2??11141? | 103??????? |
| *N. zhugeii* | ?????????? | ?????????? | ?????????? | ?????????? | ?????????? |
| *T. insularis* | 111?3?4101 | 100?000?0? | ??10?????? | 12?111141? | 1131?111?1 |
| *L. transoxiana* | ?11131?1?1 | 10??000101 | 0010000211 | ?2??11?4?? | 113??????? |
| *G. mongoliensis* | ???13????1 | 1?020????? | ?????????? | ?????1??1? | 103?1????1 |
| *B. johnsoni* | 01113131?1 | 1102000101 | 0011000211 | 1211111a11 | 11311?1100 |
| *S. gilmorei* | ?????????? | ?????????? | ?????????? | ????????11 | 113??????? |
| *T. transsylvanicus* | ?????????1 | 1?221??201 | ??1?000?11 | 12??112511 | 113??????? |
| *C. casuarius* | 112?4?3101 | 101?102211 | 11111?0210 | 122211251? | 1131111111 |
| *E. annectens* | 11214?3101 | 1022102211 | 1111100211 | 1222112511 | 1131111101 |
| *T. asinensis** | ??113??1?1 | 1?2??00101 | 001?000111 | ?????????? | ???11????1 |
| *C. agilis** | ?????????? | ?????????? | ?????????? | 12??11251? | 113?1?1??1 |
| *K. coetzeei** | ?????????? | ?????????? | ?????????? | ?????????? | 1?0?0????? |
| *L. atopus** | ?1??3????? | ?????00?01 | 1?110????? | ?2??11?3?? | 113?1????? |
| *L. arenatus** | ?????????1 | 01200???01 | 01??0001?0 | ?????????? | ????1?100? |
| *H. foulkii** | ?????????? | ?????????? | ?????????? | ?2??11?5?? | 113??????? |
| *R. suranareae** | ?????????? | ?????????? | ?????????? | ?????????? | ?????????? |
| *O. hoggii** | ?????????? | ?????????? | ?????????? | 10000112?? | ?????????? |
| *P. weishampeli** | ?????????? | ?????????? | ?????????? | 110?0112?? | ?????????? |
| *P. venenica** | ?????????? | ?????????? | ?????????? | ?????????? | ?????????? |
| *J. meniscus** | ??2??????? | ?????00101 | 0?100?1??0 | ?????????? | ?????????? |
| *C. crichtoni** | ?????????? | ?????????? | ?????????? | ?????????? | ?????????? |
| *F. tetoriensis** | ???0?011?1 | 012?0????? | ?????????? | 11?0011210 | 102???101? |
| *D. turolensis** | ?????????? | ?????????? | ?????????? | ?????????? | ???11????? |
| *O. nigeriensis** | 0110313101 | 0100002001 | 1111000210 | 1100011210 | 10011?1?11 |
| *J. yangi* | 111?2?3101 | 01???0011? | ?????????? | 11?0011?10 | 1021111111 |
| *C. leedsi** | ?????????? | ?????????? | ?????????? | ?????????? | ?????????? |
| *C. valdensis** | ?????????? | ?????????? | ?????????? | ?????????? | ?????????? |
| *D. loureiroi** | ?????????? | ?????????? | ?????????? | ?????????? | 1?0??????? |
| *E. nigeriensis** | ?????????? | ?????????? | ?????????? | ?????????? | ?????????? |
| *G. ericksoni** | ?????????? | ?????????? | ?????????? | ?????????? | ?????????? |
| NHMUKR 8676* | ?????????? | ?????????? | ?????????? | ?????????? | ?????????? |
| NHMUKR 3741* | ?????????? | ?????????? | ?????????? | ????01?2?? | ??????1??0 |
| NHMUKR 1831* | ?????????? | ?????????? | ?????????? | 11000112?? | ????1????? |

|  | 1 1 0 | 1 2 0 | 1 3 0 | 1 3 5 |
| --- | --- | --- | --- | --- |
| *L. diagnosticus* | 00000000?0 | 00000??000 | ?000000?0? | ?0000 |
| *H. foxii* | 0000000000 | 01000000?0 | 0000000?00 | 00010 |
| *M. langdoni* | ??00???0?? | ?12?0?1??? | ?00???1010 | ??010 |
| *Rhabdodon* sp. | ?????????? | ?????????? | ?0?010???? | ????? |
| *Z. shqiperorum* | 0101?????0 | 1030000??1 | 110010111? | ??01? |
| *Z. robustus* | 0?00?????0 | 11300????1 | 010010101? | ?001? |
| *T. tilletti* | 0??1000010 | 0000101111 | 0000101010 | 00010 |
| *T. dossi* | ?001?????? | 0??01010?1 | 0?0?1?10?0 | 000?0 |
| *D. altus* | 0200000010 | 0000000001 | 0000011010 | 00100 |
| *V. canaliculatus* | ?????????? | ???0000??2 | ?000011210 | 0?101 |
| *D. lettowvorbecki* | 0200?????0 | 0000000001 | 000001111? | 00101 |
| *C. dispar* | 00000?0010 | 0021001001 | 0000101110 | 00000 |
| *C. prestwichii* | ??00?????? | ?1210???0? | 1?0???1110 | 0?000 |
| *U. aphanoecetes* | 01000100?0 | 0121001??2 | 100010111? | ??000 |
| *D. lakotaensis* | ?????????? | ?????????? | ?????????? | ?0??? |
| *L. magnidens* | ?????????? | ?????11?1? | ?????????? | ????? |
| *B. dawsoni* | 100?01???1 | 2121011?1? | ?00?10131? | ??010 |
| *B. yixianensis* | 100001111? | ???10????2 | 10????1??? | 2???? |
| *H. scutodens* | 1100?????? | ?????????? | ?????0???0 | 1???? |
| *T. kerri* | ?????????? | ?????????? | ?????????? | ?0??? |
| *O. depressus** | 1????????1 | 00210????? | ?????????? | ??0?? |
| *I. fortis* | 1????????1 | 002?001?1? | ?????????? | ??0?0 |
| *P. valdearinnoensis* | ?????????1 | 0142001??? | ?1111?141? | ?001? |
| *K. katsuyama* | ?????????? | ?????????? | ?1????131? | ????? |
| *I. bernissartensis* | 2100011111 | 0132011111 | 1111101310 | 10010 |
| *M. atherfieldensis* | 1100011111 | 0031011112 | 1011101310 | 10010 |
| *A. kurzanovi* | 210001111? | 0031011112 | ?0???????? | ?00?? |
| *E. normani* | ?10??????? | ?13?0????? | ???1?0131? | ?000? |
| *H. fittoni* | ?10?01???? | ?1?10?1111 | 11?110131? | 0?01? |
| *X. yueluni* | ?????????1 | 0031011111 | 1????????? | ?1??? |
| *G. mazongshanensis* | 110??????? | ?????11??? | ?1?1?0131? | ????? |
| *P. gobiensis* | 21000111?1 | 0031011111 | 10?1101311 | 10010 |
| *E. caroljonesa* | 21000111?1 | 0031011112 | 1111101311 | 20010 |
| *J. rugoculus* | ?????????? | ?????????? | ?????????? | ????? |

|  | 1 1 0 | 1 2 0 | 1 3 0 | 1 3 5 |
| --- | --- | --- | --- | --- |
| *P. byrdi* | ???????1?? | ?????????? | ?????????? | ?0??? |
| *N. zhugeii* | ???11?11?? | ?????????1 | 11?110131? | 2???0 |
| *T. insularis* | 0??01?111? | 0051011111 | 21???????? | 2?0?? |
| *L. transoxiana* | ?????????? | ?????11??? | ??????131? | 2???? |
| *G. mongoliensis* | 1111?????0 | 0142011??2 | 11121?13?1 | 1?000 |
| *B. johnsoni* | 1111??1??0 | 0142011112 | 1112101411 | 21010 |
| *S. gilmorei* | ?????????? | ?????????? | ?????????? | ????? |
| *T. transsylvanicus* | ?????????? | ?????????? | ?????????? | ?0??? |
| *C. casuarius* | 21111?1110 | 0253011112 | 1112101411 | 21010 |
| *E. annectens* | 21111?1111 | 0253011112 | 2112101411 | 21010 |
| *T. asinensis** | 2111?????0 | 01410????? | ?1?21?141? | 2?01? |
| *C. agilis** | ?11??????? | 00420????? | 21?21?1??1 | ??010 |
| *K. coetzeei** | ?????????? | ?????????? | ?0??01101? | 0??10 |
| *L. atopus** | ???????1?? | ?????????2 | 2????????? | 2???? |
| *L. arenatus** | ???001???? | ?????11111 | 1???1????? | 1???? |
| *H. foulkii** | ???0?????? | 025?0????0 | ?112101411 | ??010 |
| *R. suranareae** | ?????????? | ?????????? | ?????????? | ????? |
| *O. hoggii** | ?????????? | ?????????? | ?????????? | ????? |
| *P. weishampeli** | ?????????? | ?????????? | ?????????? | ????? |
| *P. venenica** | ???0?????1 | 01210????? | ??01101??? | ??01? |
| *J. meniscus** | ?????????? | ?????????? | ?????????? | ????? |
| *C. crichtoni** | ?????????1 | 00110????? | ?????????? | ??0?0 |
| *F. tetoriensis** | ?????????? | ?????????? | ?????????? | ?0??? |
| *D. turolensis** | ??0??????1 | 202??11??? | ?????????? | ??0?? |
| *O. nigeriensis** | 21000111?1 | 0131011111 | 11?1101310 | 11010 |
| *J. yangi* | 110001111? | ?0310?1??1 | 1??11?131? | ??01? |
| *C. leedsi** | ?????????? | ?????????? | ?00001101? | ???0? |
| *C. valdensis** | ?????????? | ?????????? | ?0?001???? | ????? |
| *D. loureiroi** | ???????0?? | ?????????? | ?0?010111? | ???00 |
| *E. nigeriensis** | ?????????? | ?????????? | ?00001121? | ???0? |
| *G. ericksoni** | ?????????? | ?????????? | ?????????? | ?1??? |
| NHMUKR 8676* | ?????????? | ?????????? | ?1?110131? | ???1? |
| NHMUKR 3741* | ?100?????1 | 0131011112 | 111?10??10 | 1?0?? |
| NHMUKR 1831* | ???0011??? | ?????11??? | ???11???1? | ????? |
